# Macular Ganglion Cell Analysis Determined by Cirrus HD Optical Coherence Tomography for Early Detecting Chiasmal Compression

**DOI:** 10.1371/journal.pone.0153064

**Published:** 2016-04-06

**Authors:** Hae Ri Yum, Shin Hae Park, Hae-Young Lopilly Park, Sun Young Shin

**Affiliations:** 1 Department of Ophthalmology, Konyang University Hospital, College of Medicine, Konyang University, Daejeon, South Korea; 2 Department of Ophthalmology & Visual Science, Seoul St. Mary’s Hospital, College of Medicine, The Catholic University of Korea, Seoul, South Korea; LV Prasad Eye Institute, INDIA

## Abstract

**Purpose:**

To evaluate the performance of macular ganglion cell-inner plexiform layer (mGCIPL) measurement with Cirrus high-definition (HD) optical coherence tomography (OCT) for early detection of optic chiasmal compression.

**Methods:**

Forty-six eyes of 46 patients with optic chiasmal compression caused by a pituitary adenoma (PA group), 31 eyes of 31 patients with normal tension glaucoma (NTG group), and 32 eyes of 32 normal participants (control group) were enrolled. The PA group was subdivided into two subgroups, which comprised patients with temporal visual field (VF) defects (perimetric PA group, 34 eyes) and without VF defect (preperimetric PA group, 12 eyes). The mGCIPL thickness and circumpapillary retinal nerve fiber layer (cpRNFL) thickness were measured using Cirrus HD-OCT. We calculated the number of patients who had an abnormal GCA sector map, defined as at least one yellow or red sector.

**Results:**

Eyes in the perimetric PA group had significantly decreased mGCIPL thickness in all sectors. Eyes in the preperimetric PA group had significantly thinner mGCIPL in the superior, superonasal, inferonasal, and inferior sectors than eyes in control group, but no changes in cpRNFL parameters were observed. The mGCIPL thickness in inferonasal area showed the greatest AUC value (0.965), followed by the superonasal area (0.958) for discriminating preperimetric PA group from the control group. A higher reduction rate of mGCIPL thickness was noted in the nasal sector compared to other sectors, which was irrespective of temporal visual field defects. The mGCIPL thickness maps showed superonasal (P = 0.003) and inferonasal thinning in the PA group (P = 0.003), while inferotemporal thinning was revealed in the NTG group (P = 0.001).

**Conclusions:**

Macular GCIPL thickness parameters obtained with the Cirrus HD-OCT were useful in early detection of chiasmal compression and differentiating from NTG by characteristic nasal mGCIPL thinning.

## Introduction

Chiasmal compression predominantly affects crossed nerve fibers associated with the nasal hemiretina, leaving uncrossed nerve fibers that originate in the temporal hemiretina relatively preserved [1,2]. The ganglion cells on the nasal hemiretina of the fovea primarily project their axons as crossed nerve fiber to the temporal and nasal sectors of the optic nerve head (ONH) [1,2]. Chiasmal compression is traditionally diagnosed by the presence of a characteristic temporal visual field (VF) defect along the vertical meridian [2,3]. This is based on the fact that crossed nerve fibers originating in the nasal hemiretina are preferentially affected by chiasmal compression [1–3]. Recently, morphologic assessments of the optic nerve and retina have been performed in patients with chiasmal compression. An optical coherence tomography (OCT) analysis of circumpapillary retinal nerve fiber layer (cpRNFL) thickness by OCT could detect not only the characteristic cpRNFL loss corresponding to band atrophy of the optic disc in eyes with chiasmal compression but also the correlation between the degree of cpRNFL loss and the amount of visual field loss [4–9]. However, retinal ganglion cell (RGC) axon fibers originating on the nasal and temporal sides of the fovea converge at the ONH, making it difficult to identify topographical thinning patterns around the ONH using cpRNFL thickness measurements. A recent spectral domain OCT study measured macular ganglion cell thickness in patients with various types of brain lesions [10–14]. The macular ganglion cell analysis (GCA) of the Cirrus high-definition (HD) OCT (Carl Zeiss Meditec, Dublin, CA) measures macular ganglion cell-inner plexiform layer (mGCIPL) thickness within an elliptical annulus around the fovea [15]. Because the mGCIPL represents RGC cell bodies and dendrites, we would expect this analysis to effectively reveal structural abnormalities in the macular area. Therefore, we were interested in determining whether the topographical structural changes in the macular area could be visualized with mGCIPL measurements in eyes with chiasmal compression, which involves crossed nerve fibers from RGCs in nasal hemiretina.

The purpose of this study was to evaluate mGCIPL thickness measured by Cirrus HD-OCT in eyes with chiasmal compression. By comparing the mGCIPL thickness and the rate of mGCIPL thinning between eyes with chiasmal compression and normal or glaucomatous eyes, we purposed to find the specific pattern of mGCIPL thinning in eyes with chiasmal compression. Also, by comparing eyes with or without perimetric changes in eyes with chiasmal compression, the usefulness of mGCIPL evaluation in early detection of chiasmal compression was evaluated.

## Methods

This study protocol was reviewed and approved by the Institutional Review Board at Seoul St. Mary’s Hospital, part of The Catholic University of Korea College of Medicine (Seoul, Korea; KC14RISE0920). All study conduct adhered to the tenets of the Declaration of Helsinki. The institutional review board waived the need for a written consent from the participants, because of the retrospective design. Patient information was anonymized and de-identified prior to analysis.

### Subjects

We retrospectively reviewed the medical records of patients who were seen by an experienced neuro-ophthalmologist at the neuro-ophthalmology clinic of Seoul St. Mary’s Hospital between January 2009 and May 2014. Patients with a pituitary adenoma near the optic chiasm, as confirmed by brain magnetic resonance imaging (MRI), were referred from the neurosurgery clinic for evaluation of ophthalmologic problems before treatment.

A total of 46 eyes from 46 patients with a pituitary adenoma (PA), 32 eyes from 32 normal controls, and 31 eyes from 31 patients with normal tension glaucoma (NTG) were included in our analyses. Eyes in the PA group were subdivided as perimetric (34 eyes) or preperimetric (12 eyes), depending on the presence or absence of a temporal VF defect. Abnormal temporal VF defect was defined as a cluster of three points with a probability <5% on the pattern deviation map, including at least one point with a probability of <1% or a cluster of two points with a probability of <1% with respect to the vertical meridian. Only reliable VF test results (false-positive errors <15%, false-negative errors <15% and fixation loss <20%) were included in analysis. Perimetric group had to have definite temporal VF defects in both eyes. Eyes with only definite temporal VF defects in one eye or with no abnormal temporal VF defects in both eyes were classified into preperimetric group. When both eyes were eligible for study inclusion, the eye with the worse visual function was included.

Control group eyes had an intraocular pressure (IOP) < 21 mmHg, no history of increased IOP, a normal disc appearance, no visible RNFL defects and a normal VF. No ocular diseases were noted during routine ophthalmological examination of control subjects.

The inclusion criteria for patients with NTG were as follows: best-corrected visual acuity (BCVA) better than 20/40, spherical refractive error between +3.0 and -6.0 diopters, open anterior chamber angle, no tropia or phoria, and no underlying cause for optic disc damage besides glaucoma. Normal tension glaucoma was defined as the presence of an abnormal, glaucomatous optic disc (diffuse or focal thinning of the neuroretinal rim), an abnormal VF consistent with glaucoma, IOP < 21 mmHg, and an open angle (as determined with gonioscopy). A glaucomatous VF defect was defined as a cluster of three or more points with a probability <5% on the pattern deviation map in at least one hemifield with at least one point with a probability of <1%, a VF test result of “outside normal limits” in the glaucoma hemifield test, or a pattern standard deviation (PSD) with a probability of <5%. All NTG patients were matched to a PA patient by age and VF mean deviation (MD) and masked in the enrollment process during the same time enrollment period of PA patients to reduce selection bias.

### Ophthalmological Examination

All subjects underwent a medical history review and a comprehensive ophthalmologic assessment, including measurement of BCVA, refractive error, and IOP. Slit-lamp biomicroscopy, dilated stereoscopic examination of the ONH and fundus, Cirrus HD-OCT imaging (Carl Zeiss Meditec, Inc., Dublin, CA, USA), and standard automated perimetry (SAP; 24–2 Swedish Interactive Threshold Algorithm [SITA], Humphrey Field Analyzer II, Carl Zeiss Meditec) were also performed.

The Cirrus HD-OCT instrument (software version 6.0) was used to examine the macula (Macular Cube 512 × 128 protocol) and optic disc (Optic Disc Cube 200 x 200 protocol) scans to obtain mGCIPL and cpRNFL measurements. The Macular Cube protocol included the GCA, and the Optic Disc Cube protocol included the cpRNFL analysis. Image quality was assessed by an experienced examiner who was masked to patient identity and other test results. Only well-focused, well-centered, high signal strength (signal strength ≥ 7) images without eye movement artifacts were used in analyses. Average cpRNFL thickness in each quadrant (superior, temporal, inferior, and nasal) was determined for all individuals and analyzed. The GCA algorithm identified the macular RNFL and inner plexiform layer (IPL) outer boundaries. The distance between the RNFL and IPL outer boundaries was defined as mGCIPL thickness. Average, minimum, and sectoral (superotemporal, superior, superonasal, inferonasal, inferior, and inferotemporal) mGCIPL thickness and reduction rates were analyzed. Reduction rates in OCT parameters of each group were calculated from mean mGCIPL thickness in normal eyes. The reduction amount of mean mGCIPL thickness in PA group compared to those in control group was calculated as the reduction rate ([1 –(mean mGCIPL thickness of PA eye/mean mGCIPL thickness of normal eye)] x 100).

Comparisons of mGCIPL thickness measurements in the scanned patient to the age-matched, normative database are presented as color-coded maps by the Cirrus OCT. Areas of mGCIPL thickness that are found in 95% of the normal population are represented in green. Areas of mGCIPL thickness that are found in 1–5% of the normal population are presented in yellow and those that are found in <1% of the normal population are presented in red. We calculated the number of patients who had an abnormal GCA sector map, defined as at least one yellow or red sector.

We performed at least two SITA 24–2 SAP tests. The VF defect had to be present at the same location in at least two consecutive reliable VF tests for the result to be included in the study. Results from VF tests were considered reliable if fixation losses were less than 20% and false positive and false negative rates were less than 15%.

### Statistical Analysis

Statistical analyses were performed using SPSS software (version 19.0; SPSS, Inc., Chicago, IL) and MedCalc V.15.6 (Mariakerke, Belgium). Independent *t*-tests, chi-square tests, and Mann-Whitney U-tests were used to compare data from normal, PA, and NTG groups. Receiver operating characteristic (ROC) curves were used to describe the ability of OCT parameters to discriminate preperimetric PA group from healthy eyes. The DeLong method was employed to evaluate the statistical significance of differences in the AUC values. Statistical significance was defined as *P* < 0.05.

## Results

Table 1 summarizes the demographic characteristics of all study participants. There were no significant differences in age, sex distribution, refractive error, and IOP between the PA and control groups. However, the PA group had significantly worse visual acuity than both the control and NTG groups. There was no significant difference in VF mean deviation between the PA and NTG groups.

**Table 1:** Demographic data in all study participants.

		Age (years)	Male/Female	VA (log MAR)	Refraction (D)	IOP (mmHg)	Mean deviation (dB)
Control (32 eyes)		49.9±13.3	14/18	0.03±0.07	-0.97±1.63	14.69±3.49	-0.09±1.18
Pituitary adenoma (PA)							
	Perimetric group (34 eyes)	48.8±15.1	16/18	0.32±0.27	-2.24±4.69	13.85±3.03	-11.44±8.66
	Preperimetric group (12 eyes)	55.4±9.3	4/8	0.05±0.07	-0.05±0.69	13.92±2.99	-1.29±1.13
	Total (46 eyes)	50.5±14.0	20/26	0.25±0.26	-1.65±4.13	13.87±2.99	-8.54±8.65
NTG (31 eyes)		54.7±13.3	15/16	0.07±0.11	-2.59±3.34	13.03±2.18	-7.77±8.03
*P* value^a^		0.981*	0.981†	<0.001*	0.594*	0.494*	<0.001*
*P* value^b^		0.193*	0.816†	0.001*	0.305*	0.185*	0.701*

VA, visual acuity; D, diopter; IOP, intraocular pressure; dB, decibel; NTG, normal tension glaucoma. *P* value^a^; comparison between control and PA group. *P* value^b^; comparison between PA group and NTG group.

* Independent t test.

† χ^2^ test.

**Fig 1:**
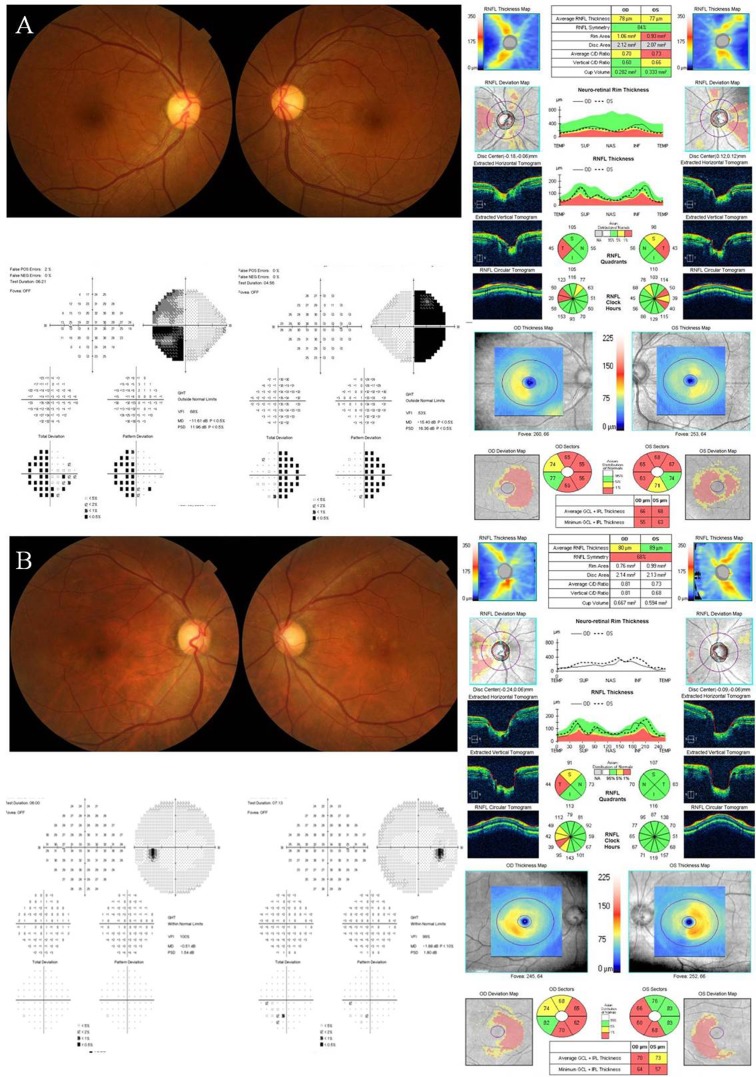
Representative cases of perimetric group and preperimetric group in patients with pituitary adenoma. (A) The patient was a 42-year old female with a characteristic bitemporal defect on visual field (VF) examination. Macular ganglion cell analysis (GCA) revealed a preferential ganglion cell and inner plexiform layer thinning in bi-nasal area in the GCA thickness, deviation and sector maps. (B) The patient was 58-year old male without any VF defects. Also, temporal circumpapillary retinal nerve fiber layer thinning was found only in his right eye. However, GCA revealed a characteristic bi-nasal pattern respecting the vertical meridian in both eyes.

Representative cases of perimetric and preperimetric PA groups are described in Fig 1. The patient described in Fig 1A was a 42 year old female who was diagnosed with a 12 x 23 x 16 mm pituitary adenoma. The patient presented with a characteristic bitemporal VF defect. Macular GCA revealed a preferential mGCIPL thinning in the bi-nasal area in the GCA thickness, deviation, and sector maps. Fig 1B shows a case of a 58 year old male with a pituitary adenoma. Results of VF testing showed no definitive abnormality in either eye. However, GCA revealed abnormalities with the characteristic bi-nasal pattern along the vertical meridian in both eyes.

### I. Macular ganglion cell analyses in normal eyes and those with chiasmal compression

**Fig 2:**
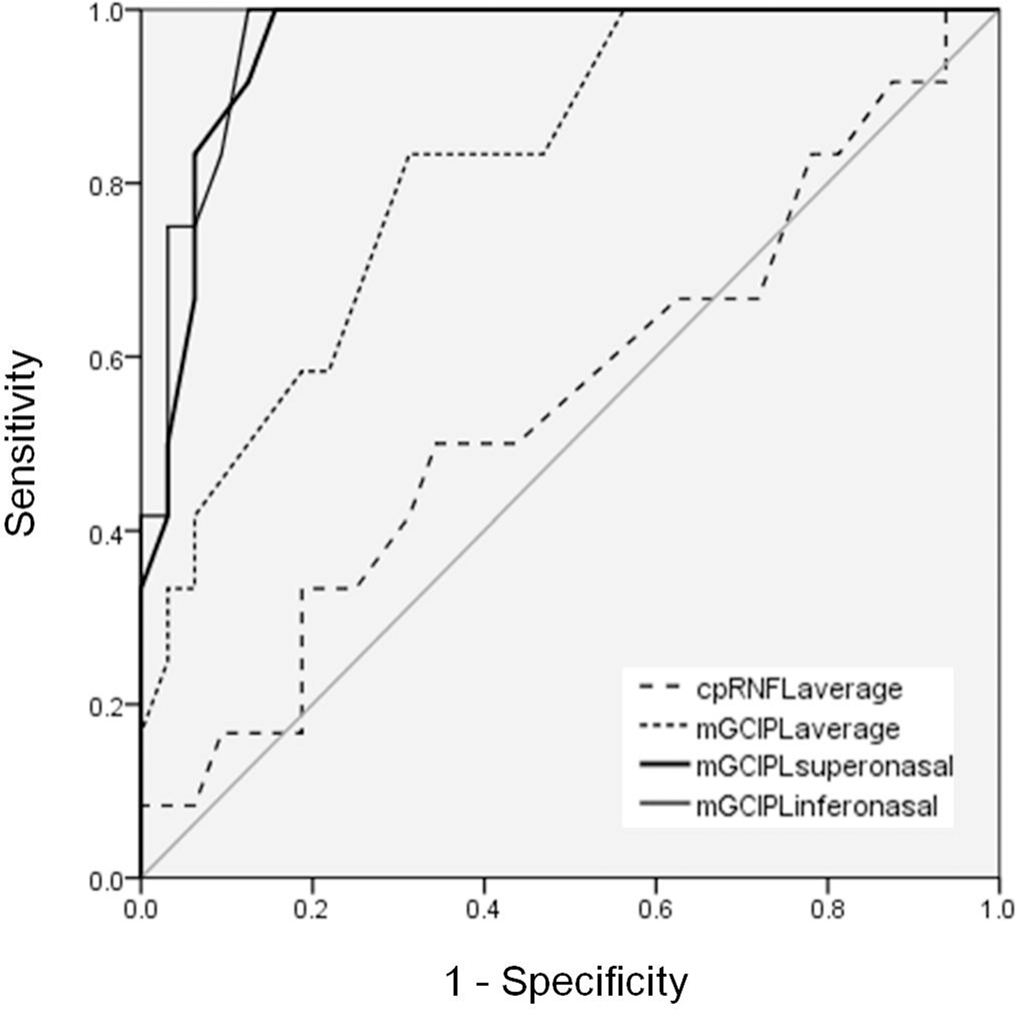
Comparison of the area under the receiver operating characteristic curves (AUC) of the macular ganglion cell and inner plexiform layer (mGCIPL) thickness and circumpapillary retinal nerve fiber layer (cpRNFL) thickness for discriminating preperimetric pituitary adenoma group from the normal control group. The AUC of the mGCIPL thicknesses in inferonasal and superonasal area were significantly greater than those of cpRNFL thicknesses (*P* < 0.001). Of the GCIPL-related parameters, the mGCIPL thickness in inferonasal area showed the greatest AUC value (0.965), followed by the mGCIPL thickness in superonasal area (0.958).

**Fig 3:**
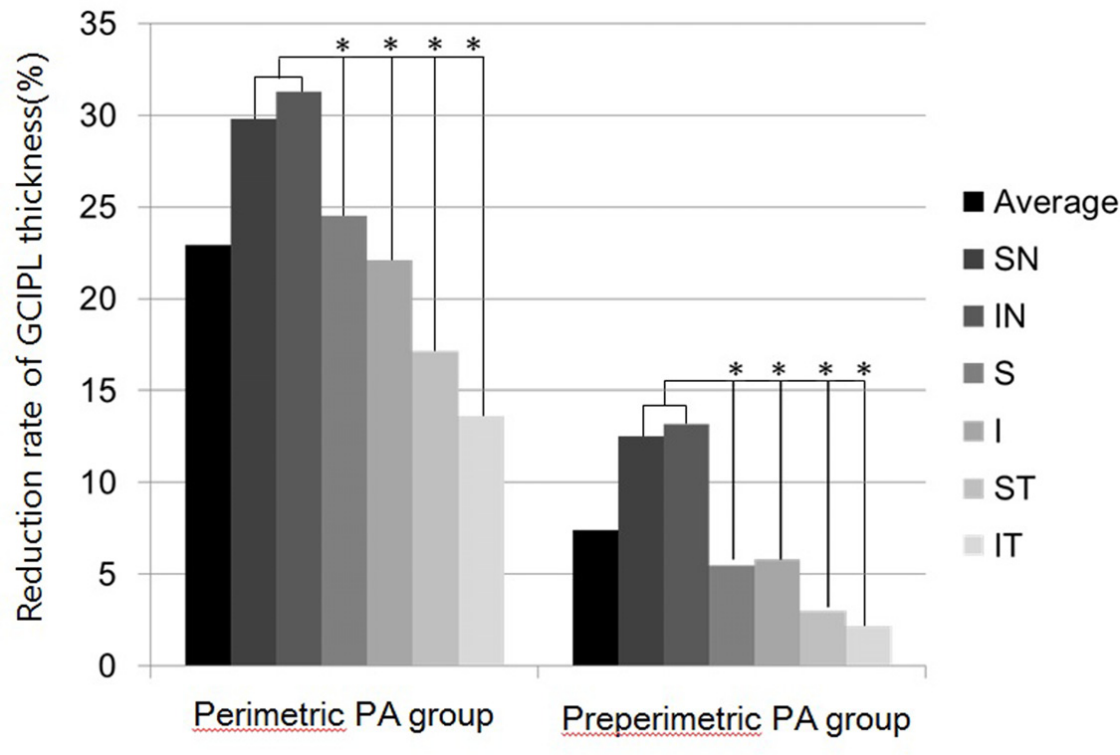
Comparison of the reduction rate of macular ganglion cell-inner plexiform layer (mGCIPL) thicknesses among sectors in the pituitary adenoma (PA) groups. The higher reduction rate of the mGCIPL thickness was noticed in nasal sector compared to other sectors, irrespective of the presence of temporal visual field defect. (*P* < 0.001) Perimetric PA eyes exhibited higher reduction rate in all sectors than preperimetric PA eyes (*P* < 0.001 for superonasal, inferonasal, and superior sectors; *P* = 0.001 for inferior sector; *P* = 0.004 for interotemporal sector).

Table 2 shows cpRNFL and mGCIPL thickness in the normal control, PA and NTG groups. Perimetric PA eyes had significantly lower cpRNFL and mGCIPL thickness in all sectors. The preperimetric PA eyes had significantly lower mGCIPL thickness compared to the control group in average thickness (*P* = 0.001), minimum thickness (*P* < 0.001), superior (*P* = 0.049), superonasal (*P* < 0.001), inferonasal (*P* < 0.001) and inferior (*P* = 0.020) sectors thickness. However, the significant changes in cpRNFL and mGCIPL thickness in the superotemporal and inferotemporal sectors were not noted in the preperimetric PA eyes. The receiver operating characteristic curves (AUC) of the mGCIPL thicknesses in inferonasal and superonasal area were significantly greater than those of cpRNFL thicknesses for discriminating preperimetric PA group from the control group (*P* < 0.001). The mGCIPL thickness in inferonasal area showed the greatest AUC value (0.965), followed by the mGCIPL thickness in superonasal area (0.958) (Fig 2 and Table 3). The reduction rates of mGCIPL thickness in all sectors in PA group are illustrated in Fig 3. A higher reduction rate of mGCIPL thickness was noted in the nasal sector compared to other sectors in both perimetric and preperimetric PA groups (*P* < 0.001). Perimetric PA eyes exhibited a higher reduction rate in all sectors than preperimetric PA eyes (*P* < 0.001 for superonasal, inferonasal, and superior sectors; *P* = 0.001 for inferior sector; *P* = 0.004 for interotemporal sector). The detection rate of abnormal color codes on GCA sector maps was analyzed according to macular region (Table 4). In the 46 patients with PA, the abnormal yellow color code was most commonly found in the inferonasal sector 34 (73.9%), followed by the superonasal 29 (63.0%) and inferior 29 (63.0%) sectors. Furthermore, analysis of the GCA sector maps revealed that 25% of preperimetric PA eyes had an abnormal yellow or red color code in the inferonasal sector.

**Table 2:** cpRNFL and mGCIPL thickness measurements in the participants.

		Control	PA group			NTG group	*P* value^a^	*P* value^b^	*P* value^c^	*P* value^d^
			Total	Perimetric	Preperimetric					
cpRNFL, μm	Average	98.44±8.08	81.72±14.96	76.47±12.96	96.58±9.24	71.19±15.60	<0.001	<0.001	0.630	0.004
	Superior	124.16±14.82	100.59±24.99	94.18±24.75	118.75±15.15	81.97±22.34	<0.001	<0.001	0.458	0.001
	Inferior	127.03±10.64	105.43±23.47	98.12±21.80	126.17±13.89	82.65±27.40	<0.001	<0.001	0.866	<0.001
	Temporal	74.06±10.04	58.50±14.02	53.88±11.77	71.58±11.64	60.90±17.46	<0.001	<0.001	0.612	0.506
	Nasal	68.38±9.58	61.91±10.18	58.88±7.87	70.50±11.37	59.06±8.57	0.006	<0.001	0.524	0.204
mGCIPL, μm	Average	84.78±4.56	68.76±10.39	65.35±9.43	78.42±6.13	67.42±9.93	<0.001	<0.001	0.001	0.574
	Minimum	83.09±4.43	60.80±13.18	56.47±11.77	73.08±8.49	58.48±15.19	<0.001	<0.001	<0.001	0.478
	Superior	85.63±5.84	68.80±13.11	64.56±12.11	80.83±7.03	68.23±12.12	<0.001	<0.001	0.049	0.845
	Superonasal	86.44±4.96	64.54±10.74	60.65±9.45	75.58±4.91	72.10±10.53	<0.001	<0.001	<0.001	0.003
	Inferonasal	84.84±4.89	62.24±10.99	58.24±9.50	73.58±5.76	69.52±9.37	<0.001	<0.001	<0.001	0.003
	Inferior	82.59±4.80	67.91±11.35	64.41±10.77	77.83±5.84	63.74±10.55	<0.001	<0.001	0.020	0.108
	Superotemporal	83.97±4.35	72.70±11.10	69.59±11.06	81.50±4.74	64.74±11.50	<0.001	<0.001	0.161	0.003
	Inferotemporal	85.34±5.23	76.24±10.35	73.71±10.54	83.42±5.35	67.90±9.61	<0.001	<0.001	0.427	0.001

cpRNFL, circumpapillary retinal nerve fiber layer; mGCIPL, macular ganglion cell and inner plexiform layer; PA, pituitary adenoma; NTG, normal tension glaucoma. *P* value^a^; comparison between normal and PA group (Independent t test). *P* value^b^; comparison between normal and perimetric group (Independent t test). *P* value^c^; comparison between normal and preperimetric group (Mann Whitney U-test). *P* value^d^; comparison between PA group and NTG group (Independent t test).

**Table 3:** Comparison of the area under the receiver operating characteristic curves (AUC) between control and preperimetric PA group.

		Area under curve (SE)	95% CI	*P* value
cpRNFL	Average	0.549 (0.101)	0.351–0.748	0.617
	Superior	0.576 (0.097)	0.386–0.765	0.445
	Inferior	0.518 (0.110)	0.303–0.733	0.854
	Temporal	0.552 (0.095)	0.366–0.739	0.598
	Nasal	0.436 (0.099)	0.242–0.631	0.519
mGCIPL	Average	0.818 (0.067)	0.687–0.948	0.001
	Minimum	0.909 (0.048)	0.816–1.000	<0.001
	Superior	0.694 (0.091)	0.515–0.873	0.050
	Superonasal	0.958 (0.027)	0.905–0.985	<0.001
	Inferonasal	0.965 (0.024)	0.917–0.991	<0.001
	Inferior	0.729 (0.078)	0.575–0.883	0.020
	Superotemporal	0.641 (0.094)	0.457–0.824	0.155
	Inferotemporal	0.579 (0.100)	0.384–0.775	0.422

cpRNFL, circumpapillary retinal nerve fiber layer; mGCIPL, macular ganglion cell and inner plexiform layer

**Table 4:** Comparison of detection rate of an abnormal color code using a GCA sector map in 46 patients with pituitary adenoma.

Location	Yellow + Red code (1% < p < 5%)			Red code (p <1%)		
	Total	Perimetric	Preperimetric	Total	Perimetric	Preperimetric
Superior, N (%)	26 (56.5)	24 (70.6)	2 (16.7)	18 (39.1)	17 (50.0)	1 (8.3)
Superonasal, N (%)	29 (63.0)	27 (79.4)	2 (16.7)	26 (56.5)	25 (73.5)	1 (8.3)
Inferonasal, N (%)	34 (73.9)	31 (91.2)	3 (25.0)	30 (65.2)	27 (79.4)	3 (25.0)
Inferior, N (%)	29 (63.0)	27 (79.4)	2 (16.7)	25 (54.3)	23 (67.6)	2 (16.7)
Inferotemporal, N (%)	13 (28.3)	12 (35.3)	1 (8.3)	9 (19.6)	8 (23.5)	1 (8.3)
Superotemporal, N (%)	24 (52.2)	22 (64.7)	2 (16.7)	18 (39.1)	17 (50.0)	1 (8.3)

GCA, ganglion cell analysis; N, number.

### II. Macular ganglion cell analysis in eyes with chiasmal compression and glaucomatous optic neuropathy

The ability of the macular GCA to differentiate between compressive optic neuropathy secondary to PA and glaucomatous optic neuropathy was investigated. A cpRNFL thinning was prominent in the superior (*P* = 0.001) and inferior (*P* < 0.001) quadrants in the NTG group compared to the PA group (Table 2). The mGCIPL thickness maps also showed superonasal (*P* = 0.003) and inferonasal (*P* = 0.003) thinning in the PA group and inferotemporal (*P* = 0.001) thinning in the NTG group (Table 2).

The detection rate of abnormal color codes using a GCA sector map shows that the inferotemporal and superotemporal sectors, which are usually involved in glaucoma, are more frequently involved in NTG group compared to PA group (Table 5). However, the superonasal and inferonasal sectors were more frequently involved in PA group, especially the superonasal sector showed statistically significant detection rate of PA group in both yellow and red color code (*P* = 0.036).

**Table 5:** Comparison of detection rate of an abnormal color code using a GCA sector map between PA group and VF- matched NTG group.

Location	Yellow + Red code (1% < p < 5%)			Red code (p <1%)		
	PA (N = 46)	NTG (N = 31)	*P* value	PA (N = 46)	NTG (N = 31)	*P* value
Superior, N (%)	26 (56.5)	18 (58.1)	0.893	18 (39.1)	14 (45.2)	0.805
Superonasal, N (%)	29 (63.0)	12 (38.7)	0.036	26 (56.5)	9 (29.0)	0.036
Inferonasal, N (%)	34 (73.9)	16 (51.6)	0.044	30 (65.2)	14 (45.2)	0.081
Inferior, N (%)	29 (63.0)	27 (87.1)	0.035	25 (54.3)	23 (74.2)	0.078
Inferotemporal, N (%)	13 (28.3)	19 (61.3)	0.004	9 (19.6)	16 (51.6)	0.003
Superotemporal, N (%)	24 (52.2)	25 (80.6)	0.011	18 (39.1)	18 (58.1)	0.102

GCA, ganglion cell analysis; PA, pituitary adenoma; NTG, normal tension glaucoma; N, number *P* Value; comparison between PA group and NTG group by Chi-square test.

## Discussion

In the current study, we confirmed that Cirrus HD-OCT GCA maps had a good ability to detect eyes with chiasmal compression. Especially, the inferonasal sector in the GCA sector map was useful in detecting eyes with chiasmal compression and showed distinct difference when compared to glaucoma group. The crossed nerve fiber pathology that occurs in eyes with chiasmal compression is efficiently revealed by characteristic nasal mGCIPL thinning using macular GCA. Our study demonstrated that macular GCA can provide valuable diagnostic information for discriminating between eyes with chiasmal compressive optic neuropathy and those that are normal or have glaucoma. Looking at the nasal sector, especially the inferonasal sector of the GCA map could aid the clinical decision to detect suspected eyes with chiasmal compressive lesion.

In this study, we were particularly interested in the diagnostic ability of macular GCA for detecting early structural changes secondary to chiasmal compression. There was typically characteristic nasal mGCIPL thinning in the pituitary adenoma patient without visual field defect. In addition, the receiver operating characteristic curves (AUC) of the mGCIPL thicknesses in inferonasal and superonasal area were greater than those of cpRNFL thicknesses for discriminating preperimetric PA group from the control group. Therefore, nasal mGCIPL thinning could be a single feature in the chiasmal compression and useful clue to necessitate brain imaging.

We were also interested in knowing whether the mGCIPL analyses could be used to differentiate eyes with chiasmal compression from eyes with glaucoma, the most common optic neuropathy in a clinical setting.

Macular GCIPL analyses more clearly visualized the characteristic topographic pattern of RGC loss than cpRNFL analyses in eyes with chiasmal compression. In this study, earlier and more prominent abnormalities in macular parameters were noted in the nasal sector than in the temporal sector. Because approximately 50% of RGCs are located within 4.5 mm of the fovea and RGC axon fibers nasal and temporal to the fovea converge on or are superimposed around the ONH [16], the characteristic topographic thinning of RGC axons can be revealed using mGCIPL measurements [12]. Color-coded mGCIPL thickness and deviation maps illustrated the preferential nasal mGCIPL thinning along the vertical meridian in eyes with chiasmal compression (Fig 1A and 1B). Interestingly, these mGCIPL findings were also observed in eyes with no VF defect (mean deviation = -1.29 ± 1.129 dB). These eyes also had a significantly thinner mGCIPL in the superior, superonasal, inferonasal, and inferior sectors than eyes in the control group like eyes with VF defect, but no changes in cpRNFL parameters were observed (Table 2). The nasal mGCIPL parameters had an AUC of 0.965 and 0.958, compared with only ranged from 0.436 to 0.576 for the cpRNFL parameters (Table 3, Fig 2). The AUC of the mGCIPL thicknesses in inferonasal and superonasal area were significantly greater than those of cpRNFL thicknesses (*P* < 0.001). In addition, a color code analysis of GCA sector maps showed that 25% of PA eyes without a definite temporal VF defect had abnormal yellow or red color codes in the inferonasal sector (Table 4). Macular ganglion cell thickness was recently suggested to be an earlier indicator of damage than VF defects in glaucomatous eyes [17–24]. A significant reduction in RGC density occurred before VF deficits were detected [25,26]. However, it remains unknown whether or not structural RGC body abnormalities precede functional changes in eyes with compressive optic neuropathy. Both structural and functional tests are important in assessing damage secondary to chiasmal compression. Our results suggest that macular GCA may allow for early detection of preperimetric compressive optic neuropathy. Identification of structural changes during early stages of compressive optic neuropathy secondary to parachiasmal tumors is crucial because a significant proportion of patients with parachiasmal tumors recover visual function following chiasmal decompression.

This study also demonstrated distinctively different patterns of mGCIPL thinning in eyes with chiasmal compression and eyes with glaucoma. Both compressive and glaucomatous optic neuropathies are generally slow, progressive processes and are accompanied by optic cup excavation and enlargement. The difficulty in distinguishing between glaucomatous optic neuropathy and compressive optic neuropathy, especially in eyes with NTG, has been previously reported [27,28]. Funduscopic characteristics such as optic disc cupping and pallor of the neuroretinal rim are considered the important hallmarks for NTG and compressive optic neuropathy, respectively. However, these are subjective and often difficult to define. In addition, compressive optic neuropathy can manifest as a glaucomatous disc cupping and visual field defect such as arcuated scotoma while some eyes with NTG show pale neuroretinal rim [29–37]. It has also been reported that up to 8% of patients diagnosed with NTG have associated compressive lesions in the anterior visual pathway [38]. In this study, examination of sectoral distributions of GCA abnormalities provides important clues for differentiating between chiasmal compression and glaucoma. In the PA group, abnormal GCA findings were most commonly located in the inferonasal and superonasal sectors (Tables 4 and 5). However, the inferotemporal sector is most commonly involved area in glaucomatous eyes, which was consistent with previous studies [18–20]. Our results illustrate the characteristic patterns of nasal-side RGC loss on macular GCA, which can alert clinicians of the possibility of a parachiasmal mass lesion and necessitate brain imaging in NTG suspects. Indications for performing computed tomography or MRI in patients with pathologic disc cupping are not uniformly accepted [31,39]. Greenfield et al. [39] reported that a younger age, lower visual acuity, vertically aligned VF defects, and neuroretinal rim pallor in excess of cupping may increase the likelihood of identifying an intracranial mass lesion. Based on our results, we recommend that preferential vertical thinning of the nasal mGCIPL is suggestive of structural abnormalities which need neuroimaging.

MGCIPL thickness analyses may be clinically useful for detecting chiasmal lesions when VF testing is unreliable or unavailable. In addition, in East Asian counties, including South Korea, where NTG is the most common form of open angle glaucoma [40–42], differentiating between glaucoma and compressive optic neuropathy is more challenging. Therefore, careful assessment of mGCIPL thickness may make correct and early diagnosis of compressive optic neuropathy more likely. Moreover, in highly myopic eyes with a tilted optic disc, also prevalent in South Korea, mGCIPL thickness measurements may be advantageous over cpRNFL thickness measurements in detecting chiasmal compression [43]. When evaluating patients with a parachiasmal tumor, nasotemporal disparities in macular GCA, which occur earlier and more prominently in nasal sectors in eyes with chiasmal compression, may be characteristic and suggestive of chiasmal compression.

Our study limitations include its relatively small sample size. Additionally, sector-based Cirrus HD-OCT GCAs are based on 6 arbitrarily-divided sectors that do not reflect the anatomical architecture of crossed and uncrossed nerve fibers in the macular area. This should be considered when interpreting sector-based macular GCA results. Because mGCIPL thickness is a new parameter that reflects RGC body thickness, further studies are needed to identify the spatial and temporal relationship between macular structural and functional abnormalities in eyes with chiasmal compression. Serial quantitative assessments of macular GCIPL thickness will provide threshold values to predict the recovery of visual function following chiasmal decompression.

In conclusion, our results show that mGCIPL thickness parameters obtained with the Cirrus HD-OCT were useful in early detection of chiasmal compression and differentiating from normal tension glaucoma by characteristic nasal mGCIPL thinning. They also provide better visualization of the characteristic vertical thinning pattern in the nasal sector. Thus, mGCIPL measurements can be used as a complementary diagnostic test when evaluating patients with early parachiasmal tumors or suspected NTG.

## Supporting Information

S1 Table cpRNFL and mGCIPL thickness measurements in the participants. (DOC)
