# Supplementary material for: Macular Ganglion Cell Analysis Determined by Cirrus HD Optical Coherence Tomography for Early Detecting Chiasmal Compression
Source: PLoS One. 2016 Apr 6;11(4):e0153064. doi: 10.1371/journal.pone.0153064 (PMC4822859; doi:10.1371/journal.pone.0153064)
Supplement: S1 Table — (DOC) [file pone.0153064.s001.doc]

| **Patient** | **No.** | **cpRNFL, μm** | | | | | **mGCIPL, μm** | | | | | | | |
| --- | --- | --- | --- | --- | --- | --- | --- | --- | --- | --- | --- | --- | --- | --- |
|  |  | **Average** | **Superior** | **Nasal** | **Inferior** | **Temporal** | **Average** | **Minimum** | **Superior** | **Supero-**  **nasal** | **Infero-**  **nasal** | **Inferior** | **Supero-**  **temporal** | **Infero-**  **temporal** |
| **Control** | 1 | 94 | 120 | 64 | 128 | 65 | 80 | 81 | 78 | 83 | 83 | 78 | 80 | 80 |
|  | 2 | 95 | 122 | 56 | 117 | 84 | 86 | 86 | 89 | 88 | 85 | 85 | 86 | 86 |
|  | 3 | 84 | 98 | 63 | 115 | 59 | 80 | 78 | 84 | 80 | 79 | 77 | 82 | 81 |
|  | 4 | 108 | 135 | 81 | 146 | 70 | 80 | 78 | 81 | 84 | 81 | 79 | 77 | 77 |
|  | 5 | 98 | 127 | 62 | 121 | 80 | 86 | 83 | 89 | 88 | 86 | 81 | 85 | 86 |
|  | 6 | 98 | 112 | 65 | 125 | 87 | 83 | 80 | 87 | 88 | 84 | 77 | 83 | 80 |
|  | 7 | 91 | 109 | 74 | 110 | 71 | 84 | 78 | 86 | 86 | 81 | 78 | 85 | 86 |
|  | 8 | 101 | 116 | 83 | 133 | 71 | 95 | 93 | 98 | 98 | 96 | 92 | 91 | 94 |
|  | 9 | 86 | 91 | 56 | 135 | 62 | 89 | 87 | 87 | 91 | 90 | 88 | 88 | 91 |
|  | 10 | 86 | 101 | 61 | 119 | 64 | 82 | 80 | 83 | 88 | 83 | 78 | 78 | 80 |
|  | 11 | 105 | 139 | 72 | 131 | 78 | 83 | 80 | 81 | 84 | 83 | 79 | 82 | 87 |
|  | 12 | 107 | 152 | 56 | 133 | 86 | 80 | 79 | 79 | 82 | 81 | 79 | 77 | 80 |
|  | 13 | 102 | 141 | 56 | 131 | 78 | 82 | 82 | 81 | 80 | 86 | 85 | 80 | 83 |
|  | 14 | 100 | 124 | 65 | 139 | 72 | 89 | 85 | 96 | 92 | 86 | 84 | 89 | 88 |
|  | 15 | 104 | 123 | 76 | 125 | 93 | 86 | 85 | 86 | 89 | 87 | 84 | 84 | 86 |
|  | 16 | 84 | 111 | 60 | 107 | 59 | 78 | 79 | 76 | 78 | 78 | 75 | 80 | 79 |
|  | 17 | 112 | 135 | 93 | 138 | 83 | 84 | 84 | 83 | 86 | 85 | 82 | 83 | 86 |
|  | 18 | 101 | 115 | 83 | 130 | 75 | 85 | 83 | 86 | 85 | 85 | 86 | 84 | 82 |
|  | 19 | 87 | 117 | 64 | 112 | 56 | 85 | 82 | 83 | 86 | 85 | 86 | 81 | 88 |
|  | 20 | 94 | 128 | 59 | 113 | 77 | 86 | 86 | 87 | 85 | 86 | 85 | 87 | 88 |
|  | 21 | 106 | 145 | 78 | 133 | 66 | 83 | 78 | 83 | 87 | 86 | 76 | 85 | 79 |
|  | 22 | 100 | 122 | 77 | 132 | 69 | 81 | 81 | 82 | 81 | 81 | 79 | 80 | 81 |
|  | 23 | 98 | 125 | 62 | 125 | 80 | 88 | 86 | 89 | 89 | 84 | 86 | 89 | 91 |
|  | 24 | 100 | 124 | 70 | 124 | 81 | 86 | 85 | 83 | 88 | 84 | 84 | 85 | 92 |
|  | 25 | 103 | 141 | 55 | 148 | 68 | 82 | 82 | 81 | 86 | 82 | 83 | 80 | 83 |
|  | 26 | 93 | 114 | 64 | 128 | 67 | 86 | 85 | 86 | 85 | 88 | 85 | 84 | 86 |
|  | 27 | 85 | 107 | 71 | 105 | 58 | 75 | 74 | 74 | 75 | 74 | 75 | 77 | 77 |
|  | 28 | 110 | 142 | 72 | 134 | 94 | 97 | 93 | 99 | 99 | 98 | 93 | 95 | 98 |
|  | 29 | 108 | 148 | 73 | 130 | 80 | 88 | 86 | 92 | 89 | 85 | 86 | 88 | 86 |
|  | 30 | 101 | 131 | 65 | 127 | 82 | 85 | 81 | 91 | 87 | 80 | 81 | 86 | 87 |
|  | 31 | 107 | 135 | 73 | 140 | 80 | 90 | 89 | 89 | 91 | 93 | 89 | 88 | 93 |
|  | 32 | 102 | 123 | 79 | 131 | 75 | 89 | 90 | 91 | 88 | 90 | 88 | 88 | 90 |
| **Perimetric**  **PA group** | 1 | 64 | 61 | 54 | 93 | 47 | 71 | 58 | 70 | 64 | 58 | 67 | 80 | 84 |
|  | 2 | 74 | 80 | 64 | 79 | 74 | 55 | 43 | 46 | 45 | 48 | 54 | 64 | 71 |
|  | 3 | 97 | 122 | 60 | 138 | 70 | 76 | 73 | 80 | 75 | 74 | 75 | 77 | 77 |
|  | 4 | 84 | 114 | 65 | 107 | 50 | 73 | 60 | 76 | 66 | 60 | 68 | 83 | 83 |
|  | 5 | 88 | 117 | 65 | 114 | 59 | 77 | 66 | 76 | 68 | 66 | 79 | 85 | 86 |
|  | 6 | 76 | 96 | 46 | 99 | 62 | 57 | 28 | 32 | 54 | 58 | 71 | 53 | 75 |
|  | 7 | 56 | 61 | 54 | 65 | 45 | 52 | 48 | 53 | 53 | 53 | 54 | 49 | 49 |
|  | 8 | 66 | 86 | 60 | 83 | 37 | 57 | 48 | 52 | 55 | 50 | 53 | 60 | 70 |
|  | 9 | 77 | 103 | 54 | 103 | 49 | 78 | 74 | 79 | 77 | 75 | 76 | 81 | 79 |
|  | 10 | 79 | 97 | 58 | 109 | 52 | 70 | 62 | 68 | 64 | 62 | 71 | 74 | 78 |
|  | 11 | 74 | 80 | 64 | 79 | 74 | 54 | 43 | 46 | 42 | 45 | 54 | 64 | 71 |
|  | 12 | 64 | 61 | 54 | 93 | 47 | 70 | 58 | 70 | 59 | 58 | 67 | 80 | 84 |
|  | 13 | 63 | 82 | 52 | 71 | 47 | 62 | 44 | 74 | 52 | 44 | 53 | 72 | 79 |
|  | 14 | 78 | 105 | 55 | 105 | 45 | 68 | 58 | 68 | 67 | 63 | 69 | 65 | 73 |
|  | 15 | 85 | 60 | 77 | 132 | 50 | 63 | 55 | 59 | 56 | 54 | 66 | 64 | 79 |
|  | 16 | 92 | 116 | 54 | 111 | 86 | 68 | 64 | 71 | 65 | 64 | 68 | 68 | 72 |
|  | 17 | 91 | 117 | 83 | 111 | 52 | 79 | 72 | 77 | 72 | 74 | 80 | 83 | 85 |
|  | 18 | 95 | 126 | 52 | 131 | 72 | 65 | 56 | 64 | 60 | 58 | 64 | 69 | 73 |
|  | 19 | 67 | 83 | 55 | 76 | 54 | 62 | 53 | 56 | 56 | 53 | 63 | 69 | 77 |
|  | 20 | 62 | 63 | 57 | 86 | 40 | 54 | 42 | 61 | 49 | 46 | 41 | 63 | 61 |
|  | 21 | 94 | 131 | 68 | 120 | 59 | 73 | 57 | 76 | 66 | 60 | 68 | 83 | 83 |
|  | 22 | 88 | 113 | 61 | 120 | 56 | 77 | 69 | 76 | 68 | 66 | 79 | 85 | 86 |
|  | 23 | 56 | 62 | 60 | 51 | 51 | 50 | 45 | 53 | 49 | 44 | 54 | 49 | 49 |
|  | 24 | 65 | 84 | 53 | 81 | 42 | 56 | 43 | 52 | 50 | 49 | 53 | 60 | 70 |
|  | 25 | 79 | 107 | 52 | 106 | 51 | 81 | 77 | 84 | 77 | 75 | 80 | 84 | 86 |
|  | 26 | 80 | 112 | 50 | 102 | 55 | 71 | 64 | 71 | 69 | 64 | 68 | 76 | 76 |
|  | 27 | 62 | 63 | 57 | 86 | 40 | 54 | 42 | 61 | 52 | 46 | 41 | 63 | 61 |
|  | 28 | 67 | 83 | 55 | 76 | 54 | 62 | 53 | 56 | 56 | 53 | 63 | 69 | 77 |
|  | 29 | 53 | 63 | 51 | 51 | 45 | 47 | 43 | 46 | 49 | 45 | 47 | 50 | 47 |
|  | 30 | 77 | 98 | 56 | 110 | 43 | 67 | 64 | 68 | 65 | 60 | 69 | 65 | 73 |
|  | 31 | 69 | 66 | 67 | 100 | 42 | 61 | 60 | 62 | 60 | 59 | 65 | 54 | 63 |
|  | 32 | 95 | 130 | 56 | 120 | 72 | 67 | 66 | 70 | 64 | 63 | 65 | 72 | 70 |
|  | 33 | 89 | 124 | 72 | 115 | 46 | 79 | 75 | 77 | 77 | 74 | 80 | 83 | 85 |
|  | 34 | 94 | 136 | 61 | 113 | 64 | 66 | 57 | 65 | 61 | 59 | 65 | 70 | 74 |
| **Preperimetric**  **PA group** | 1 | 104 | 123 | 83 | 141 | 70 | 80 | 78 | 81 | 79 | 79 | 78 | 80 | 83 |
|  | 2 | 80 | 91 | 73 | 113 | 44 | 65 | 51 | 68 | 68 | 66 | 67 | 74 | 75 |
|  | 3 | 109 | 130 | 77 | 148 | 82 | 85 | 80 | 90 | 80 | 80 | 83 | 88 | 89 |
|  | 4 | 90 | 99 | 63 | 108 | 92 | 82 | 79 | 85 | 79 | 77 | 79 | 84 | 85 |
|  | 5 | 100 | 129 | 71 | 128 | 74 | 82 | 77 | 82 | 78 | 77 | 84 | 84 | 89 |
|  | 6 | 107 | 124 | 91 | 143 | 71 | 79 | 75 | 81 | 77 | 75 | 78 | 80 | 83 |
|  | 7 | 89 | 107 | 70 | 116 | 63 | 69 | 65 | 68 | 65 | 63 | 67 | 74 | 75 |
|  | 8 | 95 | 131 | 56 | 124 | 69 | 75 | 70 | 80 | 73 | 70 | 75 | 77 | 77 |
|  | 9 | 105 | 141 | 75 | 134 | 70 | 85 | 82 | 90 | 81 | 80 | 83 | 88 | 89 |
|  | 10 | 85 | 118 | 48 | 106 | 68 | 77 | 70 | 78 | 74 | 69 | 77 | 81 | 82 |
|  | 11 | 94 | 104 | 70 | 121 | 81 | 80 | 73 | 85 | 75 | 70 | 79 | 84 | 85 |
|  | 12 | 101 | 128 | 69 | 132 | 75 | 82 | 77 | 82 | 78 | 77 | 84 | 84 | 89 |
| **NTG group** | 1 | 57 | 70 | 50 | 59 | 49 | 68 | 62 | 68 | 76 | 75 | 61 | 59 | 66 |
|  | 2 | 67 | 72 | 52 | 71 | 72 | 74 | 62 | 80 | 76 | 77 | 68 | 62 | 80 |
|  | 3 | 69 | 81 | 56 | 67 | 71 | 56 | 29 | 44 | 57 | 67 | 52 | 50 | 70 |
|  | 4 | 85 | 117 | 56 | 106 | 62 | 82 | 80 | 86 | 84 | 79 | 77 | 82 | 85 |
|  | 5 | 72 | 81 | 51 | 97 | 61 | 78 | 76 | 80 | 81 | 79 | 74 | 75 | 76 |
|  | 6 | 52 | 62 | 54 | 48 | 44 | 52 | 42 | 59 | 59 | 50 | 47 | 43 | 58 |
|  | 7 | 91 | 103 | 70 | 112 | 81 | 84 | 79 | 80 | 84 | 88 | 86 | 81 | 82 |
|  | 8 | 84 | 83 | 68 | 84 | 102 | 78 | 71 | 82 | 86 | 77 | 72 | 74 | 75 |
|  | 9 | 99 | 122 | 59 | 153 | 63 | 89 | 84 | 91 | 93 | 90 | 88 | 86 | 86 |
|  | 10 | 48 | 46 | 51 | 54 | 39 | 62 | 50 | 64 | 73 | 65 | 59 | 56 | 55 |
|  | 11 | 76 | 85 | 55 | 98 | 66 | 68 | 67 | 66 | 72 | 70 | 68 | 70 | 65 |
|  | 12 | 84 | 97 | 63 | 113 | 64 | 74 | 75 | 73 | 75 | 73 | 70 | 76 | 75 |
|  | 13 | 94 | 121 | 69 | 117 | 70 | 75 | 73 | 77 | 77 | 73 | 71 | 76 | 78 |
|  | 14 | 67 | 107 | 60 | 44 | 57 | 55 | 46 | 69 | 56 | 52 | 49 | 49 | 59 |
|  | 15 | 85 | 96 | 69 | 101 | 74 | 66 | 61 | 68 | 67 | 63 | 63 | 69 | 69 |
|  | 16 | 94 | 106 | 66 | 128 | 75 | 79 | 78 | 80 | 83 | 77 | 77 | 79 | 79 |
|  | 17 | 62 | 53 | 51 | 60 | 66 | 63 | 48 | 62 | 62 | 69 | 61 | 61 | 65 |
|  | 18 | 49 | 48 | 54 | 52 | 44 | 56 | 39 | 60 | 64 | 61 | 44 | 59 | 51 |
|  | 19 | 67 | 68 | 57 | 94 | 49 | 65 | 59 | 65 | 73 | 72 | 59 | 56 | 63 |
|  | 20 | 83 | 109 | 62 | 104 | 55 | 74 | 73 | 74 | 78 | 72 | 70 | 74 | 73 |
|  | 21 | 90 | 87 | 62 | 108 | 102 | 63 | 24 | 42 | 67 | 67 | 55 | 73 | 72 |
|  | 22 | 73 | 95 | 61 | 78 | 60 | 65 | 52 | 73 | 77 | 67 | 57 | 51 | 67 |
|  | 23 | 80 | 102 | 52 | 93 | 74 | 73 | 69 | 78 | 79 | 70 | 68 | 67 | 74 |
|  | 24 | 54 | 61 | 50 | 63 | 42 | 59 | 52 | 58 | 62 | 63 | 62 | 61 | 59 |
|  | 25 | 52 | 48 | 52 | 70 | 40 | 54 | 49 | 56 | 57 | 56 | 52 | 53 | 55 |
|  | 26 | 59 | 81 | 47 | 52 | 58 | 72 | 55 | 81 | 86 | 75 | 62 | 55 | 71 |
|  | 27 | 57 | 69 | 60 | 61 | 38 | 58 | 48 | 59 | 60 | 63 | 57 | 60 | 60 |
|  | 28 | 56 | 66 | 61 | 62 | 35 | 54 | 49 | 52 | 57 | 58 | 55 | 53 | 58 |
|  | 29 | 53 | 55 | 61 | 59 | 38 | 60 | 50 | 52 | 60 | 62 | 64 | 72 | 59 |
|  | 30 | 89 | 86 | 90 | 99 | 83 | 74 | 63 | 73 | 83 | 80 | 70 | 74 | 64 |
|  | 31 | 59 | 64 | 62 | 55 | 54 | 60 | 48 | 63 | 71 | 65 | 58 | 51 | 56 |
